# Seroprevalence and risk factors of *Toxoplasma gondii* infection in captive giant panda (*Ailuropoda melanoleuca*)

**DOI:** 10.3389/fcimb.2022.1071988

**Published:** 2022-11-28

**Authors:** Chanjuan Yue, Wanjing Yang, Xueyang Fan, Jingchao Lan, Wenjun Huang, Dongsheng Zhang, Yunli Li, Lihui Liao, James Edward Ayala, Kongju Wu, Yiyan Liu, Weichao Zheng, Lin Li, Hongwen Zhang, Xiaoyan Su, Xia Yan, Rong Hou, Songrui Liu

**Affiliations:** ^1^ Sichuan Key Laboratory of Conservation Biology for Endangered Wildlife, Chengdu Research Base of Giant Panda Breeding, Chengdu, Sichuan, China; ^2^ College of Veterinary Medicine, Sichuan Agricultural University, Chengdu, Sichuan, China; ^3^ Rare and Endangered Species Reintroduction and Species Monitoring Research Center, Schuan Academy of Giant Panda, Chengdu, Sichuan, China

**Keywords:** *Toxoplasma gondii*, giant panda (ailuropoda melanoleuea), seroprevalence, wildlife conservation, zoonosis

## Abstract

**Introduction:**

Toxoplasma gondii, a globally zoonotic protozoan parasite, infects most warm-blooded animals including the giant panda, and poses a serious threat to the giant panda conservation. However, the seroprevalence and the risk factors for toxoplasmosis in giant pandas are unknown. Here we aimed to determine the seroprevalence of *T. gondii* in the captive population of giant pandas and analyze the factors associated with the increased risk of infection.

**Methods:**

A total of 203 serum samples were collected from 157 (95 females and 62 males) captive giant pandas from 2007 to 2022, antibodies against *T. gondii* were screened using commercial ELISA and MAT kits.

**Results:**

The results showed 56 (35.67%) giant pandas were seropositive, age and transfer history between institutions were identifified as risk factors for *T. gondii* infection. It is suggested that age-related seroprevalence was the main factor, and housing multiple species in the same environment may increase the chance of cross-infection of *T. gondii*.

**Discussion:**

This study can provide research data for developing policies for the prevention and control of *T. gondii* and protecting the health of captive giant pandas and other wildlife.

## Introduction

*Toxoplasma gondii* is a globally distributed intracellular parasite capable of infecting almost all known warm-blooded animals, including giant pandas (*Ailuropoda melanoleuca*). Felines, especially feral domestic cats, are definitive/intermediate hosts of *T. gondii*, and contamination of the environment with fecal-containing oocysts from felines, is the main cause of toxoplasmosis transmission (Dubey, 2009). Toxoplasmosis can cause reproductive disorders in captive animals as well as livestock (Zhang et al., 2016), such as infertility, abortion, stillbirth, and weak fetuses, in addition, severe acute infection can even cause the death of the host (Dubey, 2022) with several infections and deaths of wildlife reported worldwide (Hollings et al., 2013; Lv et al., 2021).

The giant panda is considered a Class One protected species in China and is currently categorized as “vulnerable” by the International Union for Conservation of Nature (IUCN). One of the leading causes of death for both captive and wild giant pandas is parasitic diseases (Wang et al., 2018). It was reported that a captive giant panda in Zhengzhou zoo died of acute infection of *T. gondii* which showed that *T.* gondii can be an important factor in the health of giant pandas (Ma et al., 2015). However, to date, there are no reports about the prevalence of toxoplasmosis in giant pandas, and the major risk factors for the prevalence of *T. gondii* are also unknown. Therefore, this study aimed to estimate the seroprevalence and risk factors of *T. gondii* in captive giant pandas. Additionally, the association among age, season, and seroprevalence of *T. gondii* was investigated. Understanding the seroprevalence and risk factors associated with *T. gondii* can broaden our knowledge of the conservation of giant pandas, as well as guide us to formulate an appropriate plan for disease prevention and control within the population of this vulnerable species.

## Materials and methods

### Animal information and sampling

A total of 203 serum samples were collected from 157 (95 females and 62 males) giant panda individuals in four zoos, five breeding centers, and two nature reserves from 2007 to 2022 as part of a larger biomedical survey. One serum sample from each of the 157 giant pandas was selected for the serological investigation of *T. gondii* in this study. We classified giant pandas by the following age groups as described by Zhang and Wei (2006): cubs (0.5-2 years); sub-adults (3-5 years); adults (6-20 years); seniors (> 20 years). The dry and rainy seasons are divided according to the local statistics website of the national bureau of statistics (National Bureau of Statistics, 2022). In addition, to further study the correlation between age and the prevalence of *T. gondii*, nine adult giant panda individuals were selected from the Chengdu Research Base of Giant Panda Breeding (CRBGPB). For this subset, at least five serum samples were collected from each of the individuals during discontinuous or continuous years, with a total of 55 serum samples for this group. The nine adult giant pandas summary information was listed in **Table 1**.

**Table 1:** The summary information of the subset of nine adult giant pandas and the number of samples by year.

No.	Sex	Birth year	Source	Sample collected time			
A	Female	+/- 2006	Wild	2012	2013	2015	2017
				2018	2020		
B	Female	2005	Captive	2007	2012	2013	2013
				2018	2021		
C	Female	1999	Captive	2007	2012	2015	2016
				2018	2021		
D	Female	2007	Captive	2012	2013	2015	2016
				2018	2020		
E	Male	2004	Captive	2007	2012	2013	2015
				2016	2017	2018	2020
F	Male	2008	Captive	2015	2017	2018	2020
				2022			
G	Male	2008	Captive	2015	2017	2018	2020
				2022			
H	Male	2001	Captive	2012	2013	2015	2016
				2018	2022		
I	Female	2006	Captive	2007	2012	2013	2015
				2018	2020	2022	

**Figure 1:**
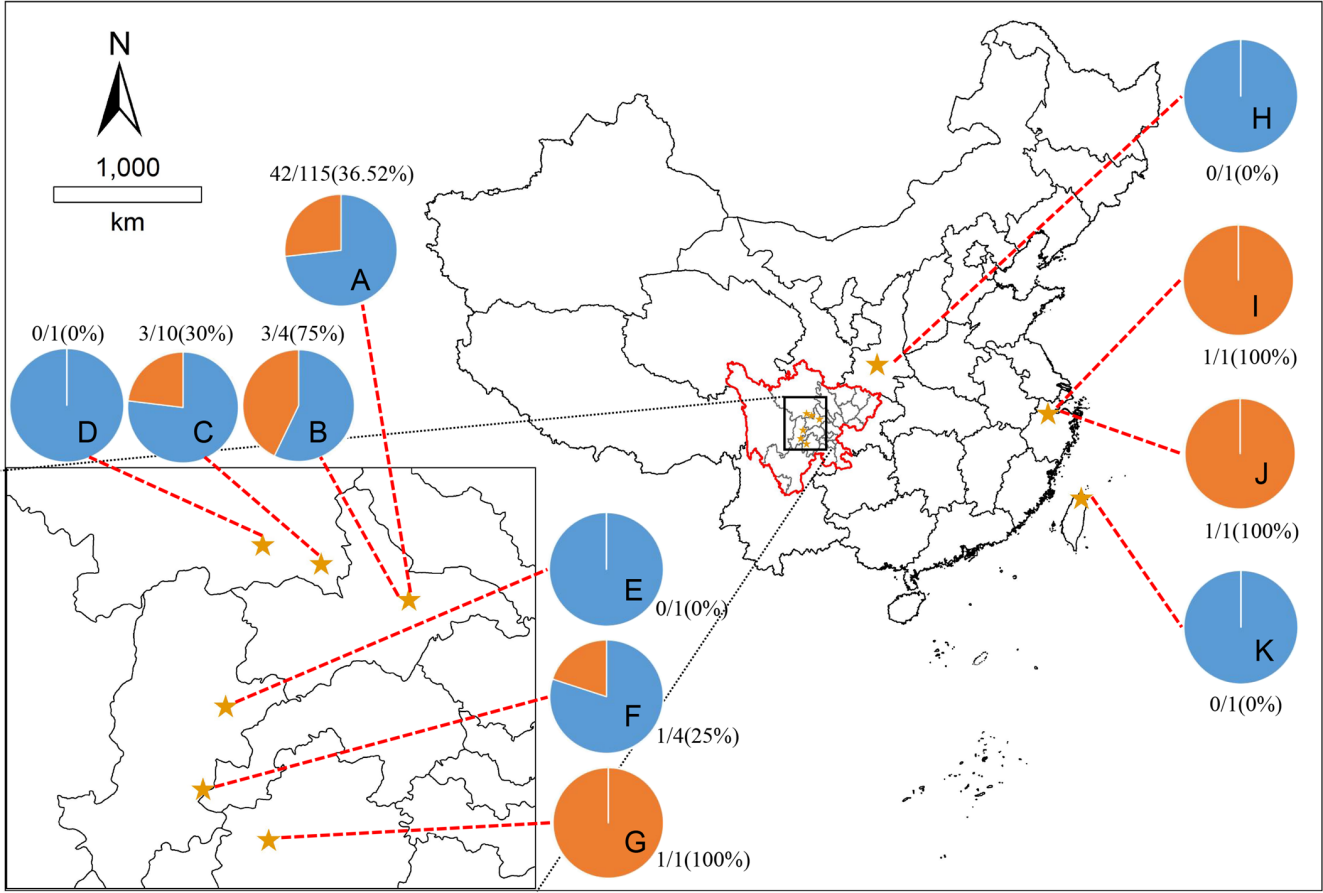
Map showing eleven sampling sites. The pie charts showed the seroprevalence of giant pandas for *T. gondii* in different sampling sites, the blue part is negative, and the orange part is positive. The locations represented by the letters: **(A)**, Chengdu Research Base of Giant Panda Breeding, Chengdu, Sichuan; **(B)**, Chengdu Zoo, Chengdu, Sichuan; **(C)**, Chengdu Field Research Center for Giant Panda-Panda Valley; **(D)**, China Conservation Research Center for Giant Panda, Wolong, Sichuan; **(E)**, China Conservation Research Center for Giant Panda, Yaan, Sichuan; **(F)**, Sichuan Daxiangling Research Base of Giant Panda Reintroduction, Sichuan; **(G)**, Heizhugou National Nature Reserve, Leshan, Sichuan; **(H)**, Shanxi Rare Wildlife Research Center Zhouzhi, Shaanxi; **(I)**, Hangzhou Zoo, Hangzhou, Zhejiang; **(J)**, Anji Bamboo Expo Park, Anji, Zhejiang; **(K)**, Taibei Zoo, Taibei, Taiwan.

This study took place in four regions of China (Sichuan, Shaanxi, Zhejiang, and Taiwan). The geographical distribution of giant pandas was as follows: 97.45% (153/157) of the samples were collected in Sichuan province; The remaining samples were collected from Zhejiang province (1/157), Shaanxi province (2/157) and Taiwan province (1/157). Among them, the largest captive population of giant pandas is in Sichuan province, with this province considered the conservation management center for the species. Therefore, the majority of the samples were collected from this region. However, the number of animals sampled per location depended on the number of transferred giant pandas (**Figure 1**).

### Sample collection

The animal handling and sampling procedures were approved by the Institutional Animal Care and Use Committee (CRBGPB) (NO. 2020006). The samples were collected from the forelimb vein of a giant panda without anesthesia, taken in the anticoagulant-free vacuum blood collection tube, and then centrifuged at 3500 rpm for 10 mins for serum separation after coagulation, and the sera were stored at -80°C after divided and labeled for the following test. All serum samples were transported to the CRBGPB, Sichuan, China, for serological testing under low-temperature storage.

### Serological analysis

A *T. gondii* MAT kit with a cut-off titer of 25 (University of Tennessee Research Foundation, Technology Transfer & Licensing, Memphis, USA) and a *T. gondii* ELISA kit (for multi-species, Haitai Biological Pharmaceuticals Co., Ltd, Zhuhai, China) were selected for detection of the giant panda *T. gondii* IgG antibody, the experimental procedure was performed according to the manufacturer’s instructions. If both two tests showed positive, the result was judged as positive, otherwise, it was negative. If the two tests showed different results, the sample needed further verification. All of the tests were performed according to the methods described in our previous study (Yue et al., 2022).

### Data analysis

Due to the small sample size of the cub age group, to better understand the relationship between the prevalence of toxoplasmosis in giant pandas, we used the following classification for statistical analysis: age (≦ 10 years old, 11-20 years old or ≧ 20 years old). We then compared these with age groups, sex (male or female), season condition (dry or rainy season) and transfer history (yes or no) were assessed using the Chi-square (χ^2^) test or Fisher’s exact test with “stats” package in R statistical software (version 4.1.2) (R Core Team, 2021). Multiple logistic regression was performed by the Wald test with the “glm” function from the “stats” package in R to assess the effect of the above five variables on the prevalence of toxoplasmosis. A probability (*p*) value <0.05 was considered statistical significance in this study. The adjusted odds ratios (ORs) and 95% confidence intervals (CIs) revealed the associations between the risk factors and the presence of *T. gondii* in giant pandas.

The McNemar Chi-square test was used to analyze the difference in agreement between MAT and ELISA tests (*P* < 0.05 was considered a significant difference). The degree of agreement between MAT and ELISA was evaluated by Cohen kappa coefficient statistics (қ), the values of қ were interpreted as follows: 0.0-0.20 = slight agreement; 0.21-0.40 = fair agreement; 0.41-0.60= moderate agreement; 0.61-0.80 = substantial agreement; 0.81-0.1 = near perfect agreement.

A total of 55 serum samples from nine adult giant pandas were used to study the correlations between age and the prevalence of *T. gondii*. The data from August was removed from the analysis since there was only one sample. Statistically significant differences were set at *p*-value < 0.05.

## Results

### Evaluation of detection methods

Following the cut-off titer provided by the commercial kits, 157 serum samples were randomly selected from giant pandas and tested using the two methods. The antibodies titer to *T. gondii* of the MAT test is shown in **Table 2**. Among the 157 serum samples, 54 (34.4%, 95% CI: 26.96-41.83) were positive by ELISA, and 56 (35.67%, 95% CI: 28.18-43.16) by MAT (**Table 3**). The degree of agreement between MAT and ELISA was calculated using the Cohen kappa coefficient (қ), the қ of ELISA showed near perfect agreement (қ = 0.97, 95% CI: 0.93-1.00), and there was no significant difference between MAT and ELISA tests (*P* > 0.05). The seroprevalence was 34.40% (95% CI: 26.96-41.83) in ELISA (**Table 3**). After repeated experiments on samples with inconsistent results of the two tests, 56 of 157 samples were finally determined as positive.

**Table 2:** Antibody titers to *T. gondii* of the MAT test.

MAT titer					Total
<25	1:25	1:50	1:100	1:200	
101	12	19	2	23	157

**Table 3:** The cross-classification and inter-agreement between MAT and ELISA for the detection of *T.gondii* antibodies in giant panda serum.

	MAT	ELISA		Total (Rate)
		+	-	
Cross-classification	+	54	2	56 (35.67%)
	–	0	101	101 (64.33%)
Total (Rate)		54 (34.40%)	103 (65.60%)	157
False negative rate (%)				0.00
False positive rate (%)				3.60
қ (95%CI)				0.97 (0.93-1.00)

χ2 = 148.45, P=0.500.

**P < 0.01, The difference is highly significant.

*0.01 < P < 0.05, difference significant.

P > 0.05, no significant difference.

қ ≤ 0.20, The degree of agreement between two methods is slight;

0.21 < қ ≤ 0.40, The degree of agreement between two methods is fair;

0.41 < қ ≤ 0.60, The degree of agreement between two methods is moderate;

0.61 < қ ≤ 0.80, The degree of agreement between two methods is substantial;

0.81 < қ ≤ 1, The degree of agreement between two methods is near perfect.

### Seroprevalence of *T. gondii* in giant panda

The results of the geographical distribution analysis showed that the seroprevalence was 32.69% for *T. gondii* of giant pandas in Sichuan province, 0% (0/1) in Shaanxi Province, 100% (2/2) in Zhejiang province, 0% (0/1) in Taiwan province; 73.25% (115/157) of the total samples were collected from the CRBGPB, and the seroprevalence of these samples was 36.52% (**Figure 1**).

**Figure 2:**
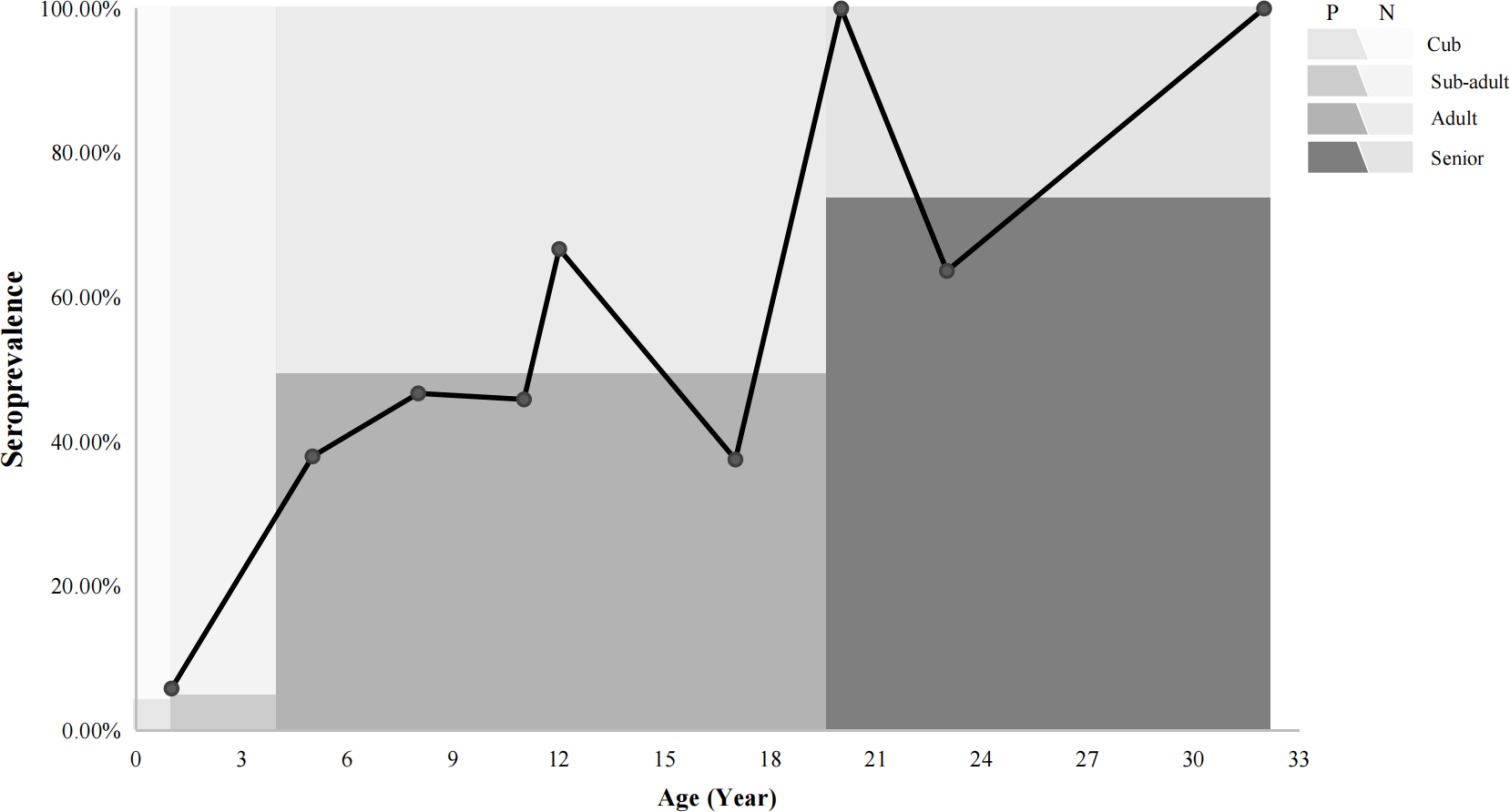
The relationships between the prevalence of giant pandas and age trends. P: positive, N: negative. The line chart shows the seroprevalence in different ages. The colors in the background indicated the percentage of the seroprevalence (The dark color: positive; The light color: negative) in different age groups.

**Figure 3:**
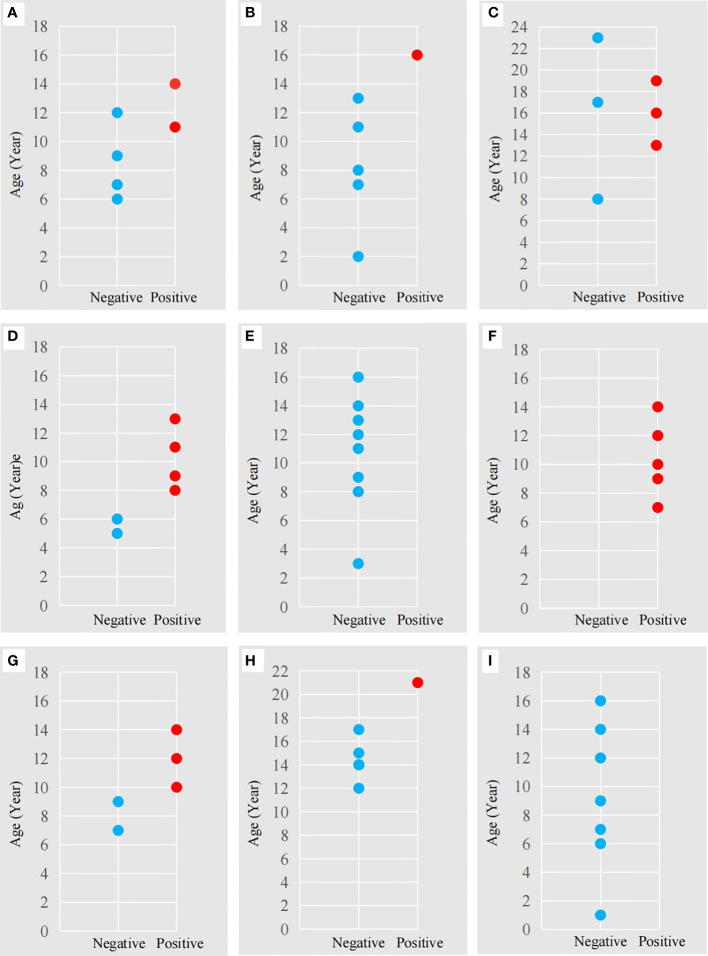
The antibodies to *T. gondii* in nine adult giant pandas at different ages. The letters **(A-I)** correspond to the nine adult giant pandas (**Table 1**) for which 55 serum samples were collected. Red dot: antibody-positive, Blue dot: antibody-negative. The abscissa represents a negative or positive result; The ordinate represents the age of the panda at the time of sample collection.

In regards to seroprevalence in different age groups, seropositivity of *T. gondii* increased with age (**Table 4**). There was an increasing trend of the seropositivity, which was highest in seniors (73.33%, 11/15), followed by adults (49.32%, 36/73), sub-adults (4.55%, 2/44), and lastly cubs (4%, 1/25) (**Figure 2**). This suggested that increasing age is one of the important influencing factors of seroprevalence of *T. gondii* in giant pandas. To further study the changing trend of antibodies to *T. gondii* by age, a subset of nine individuals for which serum samples were collected over a period of at least five years were selected. As age increased, the antibodies to *T. gondii* changed from negative to positive in most of the giant pandas (6/9, panda A, B, C, D, G, H); one panda tested positive during the entire sample period (panda F), while the other two tested negative during the sample period (panda E and panda I) (**Figure 3**).

**Table 4:** Seroprevalence and risk factors associated with seropositivity of *T. gondii* in the giant panda.

Variable	Categories	n	Seroprevalence(%)	Differences 95% (%)		P-value	OR	Differences 95% CI		P-value
				Lower	Upper			Lower	Upper	
Sex	Female	95	33.68	24.18	43.18	0.64	baseline	–	–	–
	Male	62	38.71	26.59	50.85		1.24	0.58	2.64	0.58
Age	≦10	105	22.86	30.89	14.83	<0.01	baseline	–	–	–
	11-20	37	56.76	72.72	40.90		4.31	1.90	10.06	<0.01
	>20	15	73.33	95.71	50.96		7.87	2.31	31.86	<0.01
Season	Dry season	72	38.89	50.15	27.63	0.54	baseline	–	–	–
	Rainy season	85	32.94	42.93	22.95		1.036	0.381	2.84	0.945
Transfer history	No	77	23.38	32.83	13.93	<0.01	baseline	–	–	–
	Yes	80	47.5	58.44	36.56		2.69	1.28	5.81	0.01

**Figure 4:**
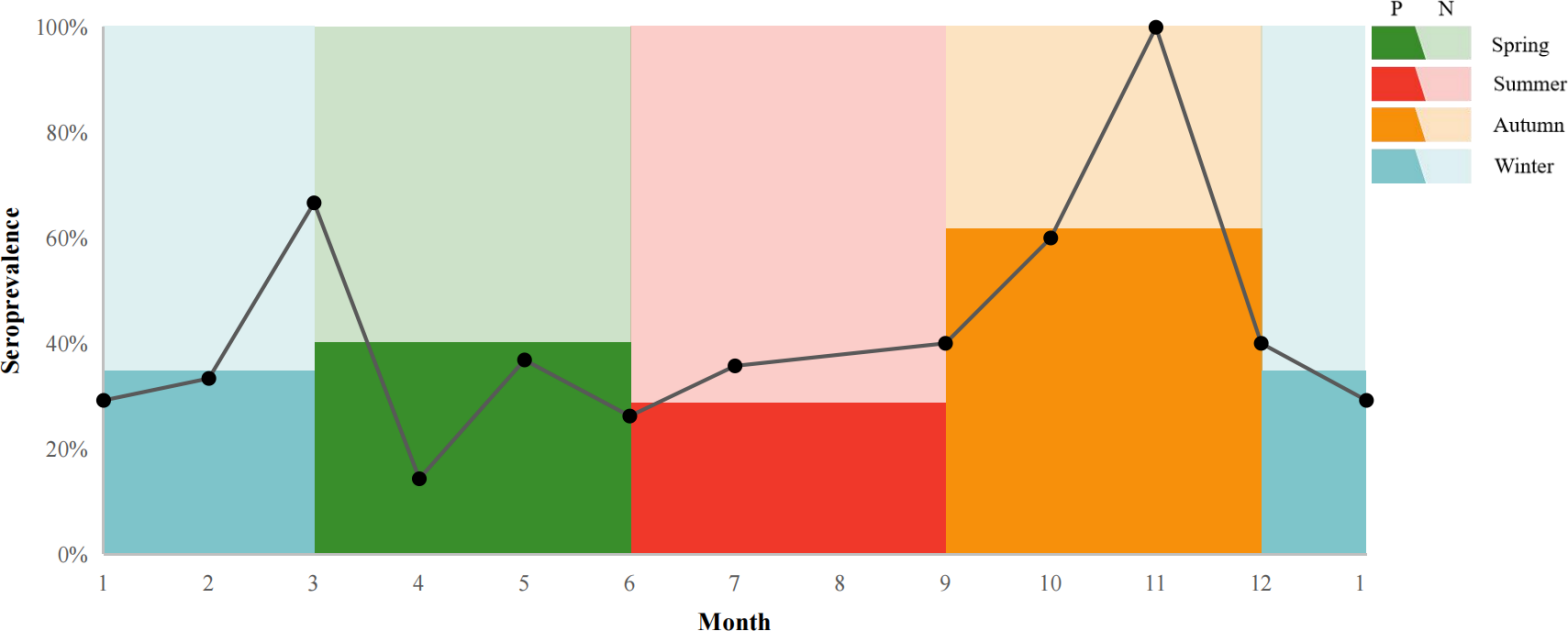
*T. gondii* seroprevalence giant pandas in different months. P: positive, N: negative. The line chart shows the seroprevalence in a different month. The colors in the background indicated the percentage of the seroprevalence (The dark color: positive; The light color: negative) in spring, summer, autumn, and winter.

The seroprevalence of *T. gondii* for giant pandas which were transferred between other institutions, was higher (47.5%, 36.56-58.44% than giant pandas without transfer history (23.38%, 13.93-32.83%) (**Table 4**). The seroprevalence of *T. gondii* in giant pandas in different months was also studied among one hundred and fifty-seven individuals. The seroprevalence of *T. gondii* in giant pandas was highest in autumn, followed by spring, winter and summer; Meanwhile, the seroprevalence of *T. gondii* reached the peak in November, and the second peak was in March (**Figure 4**).

### Risk factors

Univariate analysis of variance for the explanatory variables showed age (*p* < 0.01) and travel history (*p* < 0.01) were the two main factors strongly associated with the seropositivity of *T. gondii* (**Table 4**). However, factors like age < 10 years old, sex, and season were not classified as risk factors because the observed differences were not statistically significant. Furthermore, the results of multivariate logistic regression revealed that the >20 years of age group (OR = 7.87, 95% CI 2.31-31.86), the 11-20 age group (OR = 4.31, 95% CI 1.90-10.06), and the giant pandas that had transfer history (OR = 2.69, 95%CI 1.28-5.81) contributed to the high *T. gondii* seropositivity in this study (*p* < 0.05, **Table 4**).

## Discussion

Toxoplasmosis is a disease that results from infection with the *Toxoplasma gondii*, which can cause death in wildlife (Rouatbi et al., 2019; Wilson et al., 2021). It is also a potentially serious threat to giant pandas, which can cause reproductive disorder and even death. As infected individuals can be asymptomatic further investigation of this disease is necessary. Despite the risk of toxoplasmosis in giant pandas, there is limited data on the seroprevalence of *T. gondii* in the captive population of this vulnerable species. To the best of our knowledge, this is the first large-scale study to survey the seroprevalence and analyze the risk factors for the presence of *T. gondii* in the captive panda population. As the detection of the *T. gondii* infection in host primarily relies on serological assays, the serological methods used in this study were selected from our previous studies by comparing five commercial kits (Yue et al., 2022).

Our results showed the seroprevalence of *T. gondii* in giant pandas was 35.67%, which was consistent with Loeffler et al. (2007) (31.58%, 6/19), but was higher than Zhong et al. (2014) (1.45%, 1/69), these differences may be due to the different diagnostic methods and study designs. These similar differences were found in other wildlife studies, such as in Ursidae species. In the US, previous studies have often used ELISA or MAT to detect *T. gondii* antibodies in different Ursidae species, with the seropositivity rates of black bears (*Ursus americanus*) ranging from 14.2% to 84.5%; the brown bears (*Ursus arctos*) ranged from 24.7% to 44.0%, and polar bears (*Ursus maritimus*) ranged from 18.5% to 45.6% (Dubey, 2022).

In the present study, we found that the seropositivity of *T. gondii* increased with age. Higher infection rates were found in the senior age group, and an age-related seroprevalence was seen in giant pandas, the seropositivity became high with increased age. Moreover, the old individuals may have some underlying diseases which enhanced their susceptibility to *T. gondii* infection. This result is in agreement with previous studies (Brandon-Mong et al., 2015; Deshmukh et al., 2021). In addition, our findings demonstrated that acquired infection may be the main roution giant panda are infected with *T. gondii*. These results are consistent with previous findings in ursids (Briscoe et al. (1993), in which the prevalence of *T. gondii* in American black bears was found to increases with age.

Felines are the definitive host of *T. gondii*, infected cats can excrete oocysts into the environment, which could become a potential source of infection. Moreover, cats are popular pets in many households in both urban and rural areas, and the problem of disease transmission by stray cats has become increasingly prominent. Therefore, stray cats may be the main threat of *T. gondii* infection to the giant panda. Furthermore, rodents play an essential role in the life cycle of *T. gondii* and the epidemiology of toxoplasmosis because they are considered the primary source of infection (food and reservoir) for cats (Galeh et al., 2020). Both stray cats and rats often appear in or around the captive enclosures of giant pandas, and are difficult to control. Unfortunately, we did not collect enough data about *T. gondii* infection in stray cats and rats, so we cannot assess how serious a threat they pose to the health of captive giant pandas, this is also an important factor that needs to be investigated in the future.

As an iconic flagship species for wildlife conservation, the giant panda is considered a national treasure in China and plays a significant role not only as an umbrella species but also in national diplomacy and cultural communication. For cultural exchange, science popularization, and public education, some giant pandas will be transferred between zoos or breeding centers in different cities for exhibition. Based on our results, transferring the giant pandas between facilities was identified as one of the risk factors for *T. gondii* infection (OR = 2.69, *p* < 0.05). This result could be due to the zoos often keeping various species of animals, and as almost all known warm-blooded animals can be the intermediate host of *T. gondii*, infection rates may be higher. Therefore, managing multi-species in one area, such as in a zoo compared to breeding centers, which only have one species, may likely increase the risk of infection of *T. gondii*. Furthermore, the act of transferring giant panda or any animal is very stressful (Tang et al., 2019). This stress of moving to a new place, combined with acclimating to a new environment may reduce immune function and make the animal more vulnerable to infection.

Non-statistically significant associations were found between antibodies to *T. gondii* and sex and season. In addition, many studies showed there is no association between *T. gondii* and sex (Dubey, 2009; Must et al., 2015). The season was not an associated risk factor affecting the prevalence of *T. gondii* in giant pandas in this study. We also studied the trend of seroprevalence of *T. gondii* in giant pandas with month, and the seroprevalence was highest in November. However, other previous studies showed that the prevalence of *T. gondii* in the rainy season or monsoon associated with mild temperatures seems to be higher (Du et al., 2012), one study also mentioned the prevalence of *T. gondii* in foxes was lower at higher elevations with the cooler and drier area (Wei et al., 2021). Due to the rarity and particularity of giant pandas, the collection of serum samples is often limited and few samples in some months, such as November (three samples), resulting in the fluctuation of seroprevalence. The trend of the seroprevalence of *T. gondii* with the month in giant pandas needs further investigation.

## Conclusions

The *T.gondii* antibodies of 203 serum samples in giant pandas from 2007 to 2022 were detected and the distribution patterns of *T.gondii* infections were determined. The results showed 56 (35.67%) giant pandas were seropositive, age and transfer history between institutions were identified as risk factors for *T. gondii* infection. It is suggested that age-related seroprevalence was the main factor, and zoo housing, where multiple species are kept, may increase the chance of cross-infection of *T. gondii.* As an iconic flagship species and an umbrella species, the giant panda plays a vital role in wildlife conservation within China. It is hoped that these data can provide baseline information for developing policies and protecting the health of giant pandas and other wildlife.

## Data availability statement

The raw data supporting the conclusions of this article will be made available by the authors, without undue reservation.

## Ethics statement

The animal study was reviewed and approved by the Institutional Animal Care and Use Committee (CRBGPB) (NO. 2020006).

## Author contributions

SL, RH, and CY contributed to conception and design of the study. WY, WH, DZ, KW, and LHL collected samples. JL, XS, HZ, and YYL organized the database. XF, WY, XY, YLL and WZ performed the statistical analysis. CY, LL, and WY wrote the first draft of the manuscript. CY, SL and JA wrote sections of the manuscript. All authors contributed to the article and approved the submitted version.

## Funding

This research was supported by the Research Project of the Science and Technology Department of Sichuan Province (2020YJ0489), Chengdu Research Base of Giant Panda Breeding (2021CPB-B11, 2021CPB-B08) and National Forestry and Grassland Administration and Sichuan Province Finance Department (project title: study on main epidemiological investigation and prevention of giant panda).

## Acknowledgment

We sincerely thank the veterinary staff and keepers of the Chengdu Research Base of Giant Panda Breeding and Zoos for their participation in the sample collection work, and James Edward Ayala for reviewing the manuscript.

## Conflict of interest

The authors declare that the research was conducted in the absence of any commercial or financial relationships that could be construed as a potential conflict of interest.

## Publisher’s note

All claims expressed in this article are solely those of the authors and do not necessarily represent those of their affiliated organizations, or those of the publisher, the editors and the reviewers. Any product that may be evaluated in this article, or claim that may be made by its manufacturer, is not guaranteed or endorsed by the publisher.
